# Negative emissions in agriculture are improbable in the near future

**DOI:** 10.1073/pnas.2118142119

**Published:** 2022-03-18

**Authors:** Ronald Amundson

**Affiliations:** ^a^Department of Environmental Science, Policy, and Management, University of California, Berkeley, CA 94720

Northrup et al. (1) suggest that, “through a combination of innovations in digital agriculture, crop and microbial genetics, and electrification, we estimate that a 71% (1,744 kg CO_2_e/ha) reduction in greenhouse gas emissions from row-crop agriculture is possible within the next 15 y.” The authors (1) propose that negative emissions in row-crop farming can be achieved in less than two decades by 1) optimizing existing technologies, 2) quickly replacing existing technologies with green drop-in replacements, and 3) completely redesigning the cropping sector with improved crops, sensors, and autonomous implements. This may be a pathway to C reductions on paper, but it underestimates the yawning gap between concept and implementation.

First, the “drop-in” substitutes are not commercially available and/or have barriers to implementation. While demonstration projects or prototypes may exist, assuming that these technologies (such as electric tractors) will be in widespread application in 15 y is improbable (2). Similarly, genetic breakthroughs in crops may involve years of research.

Then there are the social, economic, and political components that must tumble into place to make the plan viable. Northrup et al. (1) present a blueprint that implicitly assumes industries, farmers, and consumers will implement it, although they do recognize farm by farm differences in objectives and possible acceptance. This leap over the social underpinnings of adoption is a serious flaw common to most negative emission projections, and is the reason that most either fail or remain unadopted (3).

The Perspective (1) also inadvertently underscores an array of enormous scientific and engineering challenges—and opportunities—that must be addressed to decarbonize society. Crop and microbial breeding, engineering of new fertilizer production methods, electrification of rural America, and the creation of circular nutrient economies are each mini-Manhattan Projects in their scope. There are presently limited incentives or infrastructure to facilitate this radical transformation of farming.

The most serious impediment is the underdiscussed Achilles’ heel of agriculture: In the 10,000 y since it was invented, it has never been generally sustainable or at steady state (4). Agriculture has always relied on borrowing resources from the future. In the past, degraded land was abandoned, and new biomes were exploited. That “extra” land is now all gone. Today, agriculture, instead, uses limited reservoirs of fossil fuel and minerals to maintain elemental steady state. Early farming altered the global C cycle and Earth’s climate system (5), and these impacts have only become magnified over time. In the 21st century, the environmental challenges of agriculture are even more complex (6). To assert this can be corrected, in decadal time scales, grossly underestimates the magnitude of the challenge. Each of the proposed “drop-in” technology replacements is an individual grand challenge for society that may possibly be alleviated over the course of the century (7). These additive, or possibly multiplicative, incremental steps may eventually coalesce over time into a whole that science aspires to. But we can’t get there, or promise that, until much work is done first.
